# Potential of the Electronic Nose for the Detection of Respiratory Diseases with and without Infection

**DOI:** 10.3390/ijms21249416

**Published:** 2020-12-10

**Authors:** Johann-Christoph Licht, Hartmut Grasemann

**Affiliations:** 1 Division of Respiratory Medicine, Department of Pediatrics, Hospital for Sick Children, Toronto, ON M5G 1X8, Canada; johann.licht@sickkids.ca; 2 Translational Medicine Research Program, Hospital for Sick Children Research Institute, Toronto, ON M5G 1X8, Canada; 3 Department of Immunology, University of Toronto, Toronto, ON M5S 1A8, Canada

**Keywords:** electronic nose, respiratory disease, respiratory infections, volatile organic compounds

## Abstract

Respiratory tract infections are common, and when affecting the lower airways and lungs, can result in significant morbidity and mortality. There is an unfilled need for simple, non-invasive tools that can be used to screen for such infections at the clinical point of care. The electronic nose (eNose) is a novel technology that detects volatile organic compounds (VOCs). Early studies have shown that certain diseases and infections can result in characteristic changes in VOC profiles in the exhaled breath. This review summarizes current knowledge on breath analysis by the electronic nose and its potential for the detection of respiratory diseases with and without infection.

## 1. Introduction

Human-exhaled breath contains over 3000 volatile organic compounds (VOCs) in gas phase, which are detectable by different laboratory methods such as gas chromatography and mass spectrometry. Exhaled VOCs include molecules such as alkanes, benzene derivatives, acetone, dimethyl sulfide, phenol, and aromatic compounds [1]. The composition of these has been found to be altered in an increasing number of medical conditions including cancers [2,3] and inflammatory bowel disease (IBD), for example [4,5,6]. With respect to the analysis of VOCs, technological advancements during recent years have resulted in the development of chemical sensing and identification devices that can capture the signatures or patterns of VOC mixtures. These ‘electronic noses’ (eNoses), mimic mammalian olfactory senses by being able to detect a ‘breathprint’ of VOC mixtures, as opposed to identifying their individual molecular constituents [1]. An eNose, as previously defined by others, is an instrument which comprises an array of electronic chemical sensors and an appropriate pattern-recognition system, capable of recognizing simple or complex odors [7]. eNoses can identify different complex odors by comparing the incoming odor with previously learnt patterns [8] by creating so called breathprints. Readings occur when VOCs react at the surfaces of the eNose sensors, causing a change in conductivity of the sensors [9]. These are then detected by transducers and converted into electrical signals that create specific VOC signatures [7]. Several distinct eNose technologies have been developed. These include the Aeonose, which uses micro hotplate metal-oxide sensors [9], the BIONOTE eNose based on QCM sensors utilizing anthocyanin-coated gold electrodes [10,11], the Cyranose 320, using a carbon black-polymer sensor array [12], the Tor Vergata eNose, using quartz crystal microbalances (QCM) covered with metalloporphyrins [13], the Common Invent eNose using metal oxide semiconductor sensors [14], the Owlstone Lonestar eNose based on field asymmetric ion mobility spectrometry [15], and the SpiroNose, using cross-reactive metal-oxide semiconductor sensors [16]. eNoses detect mixtures of VOCs to create breathprints—they do not generally identify individual molecular compounds. The use of other analytic methods, primarily gas chromatography-mass spectrometry (GC-MS), being used in parallel or in addition to eNose measurement, are being explored to help identify the specific biomarkers responsible for the changes in breathprints and to test the eNoses for accuracy in detecting certain conditions. The use of eNose plus GC-MS will be contextualized in more detail in subsequent chapters.

Detection of VOCs in exhaled air is showing immense promise for improving diagnostic and screening standards of certain lung and airway diseases. Lung cancer is among the most commonly diagnosed malignancies and also among the leading causes of death worldwide [17]. In lung cancer, common methods of detection—imaging by chest radiography (X-ray), computerized tomography (CT) or magnetic resonance imaging (MRI), and bronchoscopy—carry many limitations from a mass screening perspective [3]. Interestingly, recent studies have shown that certain VOCs, including isopropanol, acetone, pentane, and benzene can serve as biomarkers for lung cancer [18,19]. Various eNose models have been used to date, to discriminate breathprints of lung cancer patients from those of healthy subjects [18,20,21,22,23]. Positive findings were supported with robust, reproducible data consistent across several groups, and have also shown differentiation of lung cancer from other respiratory diseases [18,21,24,25,26], suggesting that eNoses could be used as clinical screening tools. In addition, there is early evidence to show that VOC patterns detected by eNose can also predict response to novel cancer treatments, as demonstrated in patients with advanced non-small cell lung cancer and anti-programmed cell death 1 (anti-PD-1) immunotherapy [16]. The potential of eNose technology in the detection of lung cancer will be addressed in more detail below.

Electronic noses were developed for olfactory analysis in commercial settings such as food quality control, environmental monitoring, military purposes and, more relevant for this review, also in areas of research with focus on diagnosis of disease [27]. With regards to potential relevance for infectious diseases, biosensors have proven to be an effective method to sense foodborne pathogens, such as *Salmonella* contaminating packaged meat [28]. As early as in 1997, Gibson et al., reported detection of certain microorganisms from plate cultures [29] and in 2004, Pavlou et al., found that an eNose could detect *Mycobacterium tuberculosis* (TB) in human sputum [30]. A 14-sensor conducting polymer array eNose discriminated between *M. tuberculosis, M. avium, M. scrofulaceum*, *P. aeruginosa* cultures, and non-infected control samples in vitro. Using principal component analysis (PCA), 100% of TB cultures were identified and discriminated from other bacterial cultures [30]. In a study comparing healthy subjects and tuberculosis patients, an eNose discriminated the two groups with high sensitivity and specificity of 95.9% and 98.5% respectively [31]. Furthermore in 2006, Thaler and Hanson showed that patients with bacterial rhinosinusitis, caused by either *Staphylococcus aureus* (SA) or *Pseudomonas aeruginosa* (PA), could be distinguished by eNose from patients without infection, allowing for correct diagnosis in 72% of cases [32].

With advances in technology, there is now accumulating evidence that eNoses have the potential to address significant unmet clinical needs in both discrimination of one disease from another, as well as in the timely detection of airway infections in patients with underlying respiratory diseases at the point of care (POC) [33,34,35]. In recent years, eNoses have advanced in their discriminative accuracy from being able to detect differences between specific disease groups, to achieving similar results as diagnostic tests such as exhaled nitric oxide (F_E_NO) and pulmonary function testing for asthmatics [36]. As this technology is being developed and investigated in comparison to established tests, it is important to critically examine factors that may influence its diagnostic performance across disease groups. In this review, we will summarize current knowledge relevant to the potential roles of eNose technologies in respiratory diseases including lung cancer, asthma, COPD, cystic fibrosis (CF), primary ciliary dyskinesia (PCD), and non-CF bronchiectasis.

## 2. eNose Technology in Respiratory Disease and Infection

### 2.1. Lung Cancer

Late diagnosis of lung cancer contributes to its high lethality; only about 15% of patients are diagnosed with early stage disease, five-year survival rate is low, and over half of all lung cancer patients die within one year of diagnosis [37,38]. There is an unmet need for simple, affordable, and accessible innovative tools for the (early) detection of lung cancer, and eNose technology has emerged as such a tool. Tirzīte et al., utilized a Cyranose 320 eNose to compare breath profiles of 252 lung cancer patients to those of 223 patients without cancer [38]. Cancers included squamous cell cancer, adenocarcinoma, undifferentiated non-small cell lung cancer, small cell lung cancer, and large cell lung cancer [38]. Non-smokers and smokers with or without lung cancer were compared. 128/133 cancer patients who were non-smokers and 114/119 of those who were smokers were diagnosed correctly by eNose (sensitivities of 96.2% and 95.8%, respectively) [38]. In a similar study, van de Goor et al., using an Aeonose device in 60 lung cancer patients and 107 healthy controls, obtained a diagnostic accuracy of 83% with a sensitivity of 83% and specificity of 84% [9]. The study included small cell and non-small cell lung cancer patients. Here, the authors suggested to utilize the eNose in combination with low-dose CT scans, with the aim of reducing false-positive results by CT imaging alone [9]. McWilliams et al., in an earlier study, utilized a Cyranose 320 to discriminate lung cancer patients from high-risk control subjects [26]. Exhaled breath from 191 subjects including 25 with lung cancers and 166 high-risk smokers were analyzed by a Cyranose 320. Patients with squamous cell carcinoma, adenocarcinoma, small cell lung cancer, and non-small cell lung carcinoma were included [26]. VOC breathprints could discriminate lung cancer patients from high risk controls with >80% accuracy [26]. Interestingly, a cheaper, alternative eNose technology called BIONOTE, which differs from the Cyranose and Aeonose in its working principle, sensing material, sensor array composition, and molecular selectivity, produced similar results [11]. In this study, 100 high-risk individuals participating in a screening program for lung cancer were included. Cancers identified included squamous cell carcinoma, adenocarcinoma, and undefined lung cancer [11]. Partial least square discriminant analysis (PLS-DA) [39] was used for analysis of the eNose data. BIONOTE sensitivity and specificity were reported at 86% and 95%, respectively, with an area under the receiver operator characteristic curve (AUROC) of 0.87 [11].

Frequent screening tests are of great importance for individuals exposed to asbestos because of the lifetime increased risk for malignant pleural mesothelioma (MPM). In a study on detection of MPM, Lamote et al. utilized a combination of four different eNoses—Cyranose 320, Tor Vergata eNose, Owlstone Lonestar eNose, and Common Invent eNose—and GC-MS [40]. The aim of this cross-sectional, case-control study was to investigate the accuracy of eNose and GC-MS in discriminating healthy controls (*n* = 16), asymptomatic asbestos-exposed subjects (AEx, *n* = 19), patients with benign asbestos-related disease (ARD, *n* = 15), and MPM patients (*n* = 14). Data were analyzed using AUROC graphs, and the final eNose breathprints were established by merging the sensor data of all four eNoses [40]. GC-MS and eNose differentiated MPM from healthy controls with 71.4% and 65.2% accuracy, MPM vs. AEx with 97.0% and 73.1% accuracy, and MPM vs. AEx + ARD with 93.8% and 73.7% accuracy, respectively [40]. Thus, in this study GC-MS outperformed eNose by >20% accuracy in discriminating between MPM vs. HC and MPM vs. AEx + ARD. Nevertheless, these findings are still promising as the main advantages of eNoses are ease of use and accessibility, as well as lower costs. Further developments and improvements of eNose devices and a combination of eNose and GC-MS technologies should be explored to further improve detection accuracy of various malignancies. As illustrated by Lamote et al., implementing these or similar modalities could make screening of asymptomatic, high-risk individuals faster and more cost-effective, which may allow for earlier interventions leading to improved management and clinical outcomes.

### 2.2. Asthma

Diagnosing asthma, as well as differentiating between eosinophilic, neutrophilic or other asthma endotypes, can be challenging. Dragonieri et al. investigated whether people with an established diagnosis of asthma could be discriminated from controls by eNose, and whether different degrees of asthma severity could also be identified. Subjects inspired VOC-filtered air by tidal breathing for 5 min, and a single expiratory vital capacity was collected into a Tedlar bag, which was subsequently sampled by a Cyranose 320 [41]. Based on individual’s breathprints, the Cyranose was able to separate mild asthma from controls. Patients with mild asthma could also be distinguished from those with severe asthma, though less distinctly (cross-validation value (CVV) of 65%) [41]. Plaza et al., performed a cross-sectional proof-of-concept study comparing VOC breathprints in different asthma subtypes [42]. Exhaled air from 52 patients with persistent asthma was analyzed by a Cyranose 320. Eosinophilic, neutrophilic, and paucigranulocytic inflammatory asthma phenotypes were characterized by inflammatory cell counts in induced sputum. Breathprints were significantly different in eosinophilic compared to both neutrophilic (accuracy 73%, *p*-value = 0.008, AUROC 0.92), and paucigranulocytic asthma (accuracy 74%, *p*-value = 0.004, AUROC, 0.79), and neutrophilic was different from the paucigranulocytic phenotype (accuracy 90%, *p*-value = 0.001, AUROC 0.88), supporting the concept of using an eNose as an alternative to sputum cytology. Plaza et al.’s observations were consistent with similar studies. Ibrahim et al., reported an 83% accuracy discriminating eosinophilic from non-eosinophilic asthma, and 72% for distinguishing neutrophilic from non-neutrophilic phenotypes, using GC-MS to detect exhaled VOCs [43]. Wagener et al., also used an eNose to differentiate eosinophilic from non-eosinophilic asthma breathprints in 27 patients with an accuracy of 85% and AUROC of 99% [44]. Interestingly, a similarly high accuracy was found by van der Schee et al., in predicting the response to corticosteroid therapy in 25 asthma patients. eNose was more accurate than sputum eosinophil counts (AUROC 0.883, *p*-value = 0.008 vs. AUROC 0.610, *p*-value = 0.441 respectively) or F_E_NO (0.545, *p*-value = 0.751) [45]. In further support of the above findings, exhaled breath samples from adults with severe asthma of the “U-BIOPRED” (Unbiased Biomarkers for the Prediction of Respiratory Disease Outcomes) cohort were used in a longitudinal multicenter study by Brinkman and colleagues [46]. Here, severe asthma phenotypes were assessed over time using both clinical characteristics and exhaled metabolomic breathprints, revealing three eNose-derived disease clusters (*n* = 26/33/19). A four-eNose panel was used, including the Tor Vergata, Cyranose 320, Owlstone Lonestar, and Common Invent eNose. At baseline and at 12–18 month follow-up visits, F_E_NO, spirometry, and induced sputum marker values were obtained. Asthma patients falling into each of these clusters showed differing clinical characteristics, such as systemic inflammatory markers, circulating eosinophil and neutrophil counts, and oral corticosteroid use. These data supported the notion that exhaled VOCs in asthma may be associated with systemic and local eosinophilic inflammation and may help to close the gap between clinical and laboratory tests in phenotyping severe asthma [46]. There is also evidence that the eNose can distinguish patients based on their current level of asthma control. In a recent cross-sectional study by Tenero et al., 28 children with asthma were categorized into controlled (*n* = 9), partially controlled (*n* = 7), or uncontrolled (*n* = 12) groups [47]. A Cyranose 320 discriminated between healthy controls (*n* = 10) plus controlled asthma (non-symptomatic) and partially-controlled plus uncontrolled asthma (symptomatic) with an AUROC of 0.85, and a sensitivity and specificity of 0.79 and 0.84, respectively [47].

eNose technology for asthma diagnosis and phenotyping also showed promising results when compared to conventional testing methods. Montuschi et al., compared the diagnostic accuracy of a Tor Vergata eNose, F_E_NO, and pulmonary function testing. Twenty-seven patients with intermittent or mild persistent asthma and 24 healthy subjects were studied. Exhaled breath was collected in Tedlar bags following a 2 h period of fasting. GC-MS was performed to confirm differences in VOC patterns between groups, and to confirm that exhaled breath samples remained stable within 48 h from collection [36]. eNose alone was able to discriminate between asthma and healthy controls in 87.5% of cases, outperforming F_E_NO (79.2%), spirometry (70.8%), and the combination of F_E_NO and spirometry (83.3%). The combination of eNose analysis of exhaled alveolar air with F_E_NO had the highest diagnostic accuracy for asthma (95.8%) [36]. No correlation was found between the eNose results, F_E_NO, and lung function in asthma or healthy controls [36].

Bannier et al. investigated the potential of an eNose for accurate diagnosis of lung disease by comparing patients with asthma, CF, and healthy controls [48]. This cross-sectional study in children 6 years of age or older included 20 with moderate to severe asthma, 13 with an established diagnosis of CF, and 22 healthy controls [48]. Asthma was defined as presenting with typical respiratory symptoms in combination with reversible airways obstruction on pulmonary function testing [49]. Almost all children enrolled (54/55) were able to perform the measurements. An Aeonose eNose showed high accuracy in differentiating asthma from CF (AUROC 0.90, sensitivity 89%, specificity 91%) and CF from controls (AUROC 0.87, sensitivity 85%, specificity 77%), while the accuracy was lower when discriminating asthma from healthy controls (AUROC 0.79, sensitivity 74%, specificity 91%) [48]. Discrimination between different diseases, i.e., asthma and CF, showed similar results to a report by Fens et al. for adults with asthma or COPD (88%) [50]. This study did not account for different subtypes of asthma and was limited by a relatively small sample size.

Finally, Brinkman et al., utilized eNose in combination with GC-MS to differentiate between stable and unstable episodes of asthma [51]. A panel of four eNoses was again used, for which the data were merged to produce a final, combined breathprint. 23 patients with mild to moderate asthma were included and exhaled breath profiles measured at baseline, loss of control, and recovery. PCA of eNose data showed 95% distinction between asthma at baseline and at loss of control, and 86% between loss of control and recovery. In comparison, GC-MS data showed much lower classification accuracies of only 68% for baseline vs. loss of control, and 77% for loss of control vs. recovery [51]. GC-MS detected exhaled metabolites that were significantly associated with sputum eosinophils. This study is one of the first to compare these two VOC-detecting technologies to longitudinally monitor exhaled breath profiles during worsening and subsequent recovery of asthma control. Three specific compounds of interest, methanol, acetonitrile, and bicyclo [2.2.2]octan-1-ol, 4-methyl were identified by GC-MS [51]. Interestingly, the composite eNose technologies were superior in their discrimination between controlled and uncontrolled asthma, when compared to GC-MS, but eNose findings did not correlate with sputum eosinophil and neutrophil percentages, which was different from the GC-MS results [51]. This may indicate a potential advantage of using both detection strategies together. The advantage of the eNose lies in detecting smaller changes in exhaled VOC profiles that may not be detected by GC-MS (i.e., broader sensitivity), whereas GC-MS has the ability to pick up more specific biomarker signals associated with changes in local inflammation during asthma flare-ups [51].

### 2.3. Chronic Obstructive Pulmonary Disease

Chronic obstructive pulmonary disease (COPD) and asthma are both common and despite the fact that they are different disease entities, there can be significant clinical overlap between the two. The potential for accurate diagnosis of COPD and discrimination from asthma by exhaled breath profiles was first studied by Fens et al. [50]. This cross-sectional study included 21 asthmatics with fixed and 39 with reversible airways obstruction, as well as 40 patients with a diagnosis of COPD. While asthma with reversible or fixed airway obstruction could not be distinguished based on breathprints, both asthma with fixed obstruction and asthma with reversible airway obstruction were significantly different from COPD (accuracy of 88% and 83%, respectively) [50]. These findings suggested that eNose may represent a diagnostic option for patients having overlapping symptoms between fixed-obstruction asthma and COPD. In addition to discriminating from asthma, recent data suggested that eNose technology may be able to detect flare-ups or exacerbations of COPD (ECOPD). An ECOPD is characterized by a burst of pulmonary and systemic inflammation, and is usually the result of bacterial or viral infection [52,53]. These events can significantly influence disease progression as well as morbidity [54] and mortality [55]. Potential pathogenic micro-organisms (PPMs) in sputum or bronchoalveolar lavage (BAL) are only identifiable in up to 50% of patients experiencing an ECOPD [56,57]. Shafiek et al. utilized a Cyranose 320 to discriminate between infectious vs. non-infectious ECOPD or pneumonia, and showed differences in VOC breathprints [33]. Among ECOPD patients, the eNose could discriminate infected vs. non-infected COPD patients with a 75% success ratio, 88% sensitivity, and 60% specificity [33]. These findings may allow for a novel strategy in diagnosing ECOPD associated with bacterial infections in routine clinical practice, rather than depending solely on clinical diagnosis.

There are several indexes of COPD severity and disease progression, including the six-minute walk test distance (6MWD), body mass index (BMI), airflow obstruction, dyspnea, and exercise (BODE), that can be used to assess the functional status of COPD patients. Since many of these tests are limited by patient compliance, space and time (e.g., availability of a 30 m hallway to perform 6MWD), Finamore et al. investigated whether VOC analysis by eNose could predict the functional status and its variation over time in COPD patients [58]. In this monocentric prospective study with one-year follow-up, patients performed pulmonary function testing, arterial blood gas analysis, bioimpedance, 6MWD, and VOC analysis by eNose in 63 patients. A BIONOTE eNose was used, and partial least square discriminant analysis (PLS-DA) to calculate outcomes-predictive accuracy, sensitivity, and specificity [58]. The eNose predicted BODE scores with 86% accuracy, and quartiles of normalized 6MWD (n6MWD) with 79% accuracy. Reference quartiles of n6MWD to the Global Initiative for Chronic Obstructive Lung Disease (GOLD) classification were as follows: 22–111 m/m^2^ corresponds to GOLD class A, 112–145 m/m^2^ to B, 146–165 m/m^2^ to C, and 166–215 m/m^2^ to D (quartiles 1–4) [58]. A change in n6MWD after one year by more than the median value of decline was predicted with an accuracy of 86% by eNose vs. 52% by GOLD classification alone, and 78% by both measures combined. These data supported that eNose technology could be further developed as a simple, and inexpensive tool to assess COPD functional status. To tangibly illustrate costs, in this study for example, an eNose analysis vs. a 6MWD represented a difference of €10 vs. €50, respectively [58].

Finally, van Velzen et al. performed a randomized controlled trial measuring exhaled breath profiles in COPD patients with and without exacerbations over a period of three years, comparing eNose and GC-MS [59]. This study included 31 patients with COPD exacerbations and 37 with stable COPD, and found significant differences between breath profiles of these patients. eNose discriminated patients with stable disease from ECOPD with an accuracy of 75%, which was very similar to GC-MS (71%) [59]. GC-MS analysis yielded ten compounds of significance in discriminating between the two groups. Similar to Brinkman et al. [46], this study utilized a panel of four eNoses, for which the data were merged. The Common Invent eNose drove the discriminative signal in detecting exacerbations most definitively [59]. The similar accuracies in detecting stable and exacerbated COPD states by these technologies is encouraging. In their work, Van Velzen et al. highlighted their approach of GC-MS and eNose technologies complementing one another: GC-MS can identify specific compounds needed to inform the fine-tuning of metabolite-specific sensor arrays on an eNose, for more precise recognition of disease-specific VOC profiles [59]. This or similar types of combined approaches will hopefully accelerate the improvement of eNose technologies to the point where they can be used as diagnostic point-of-care tools in clinical practice.

### 2.4. Cystic Fibrosis, Bronchiectasis and Primary Ciliary Dyskinesia

Lung disease in cystic fibrosis (CF) and primary ciliary dyskinesia (PCD) share similarities as both are genetic diseases associated with neutrophil-dominated airway inflammation, recurrent and chronic bacterial infections, retention of suppurative airway secretion, development of bronchiectasis, and chronic loss of lung function [60,61,62]. In CF, airway secretions are dehydrated due to water/electrolyte imbalance; secretions become difficult to clear and provide optimal conditions for bacterial infections. In PCD, defective ciliary motion leads to disturbed mucociliary clearance, which also results in recurrent and persistent sinorespiratory infections [62]. Although similar in their clinical presentation, CF and PCD are different entities, and several studies have shown differences in VOC breath profiles between CF and PCD [35,63]. eNose technology has also been able to detect differences based on bacterial colonization and disease exacerbation in these diseases. In a study of 50 children, 25 with CF and 25 with PCD, Paff et al. could show that breathprints from healthy controls differed from both CF and PCD (AUROC of 0.76 and 0.80 respectively), and PCD differed from CF as well (AUROC of 0.77) [64]. The authors speculated that distinct inflammatory and metabolic processes in CF or PCD airways would generate different volatile metabolites, and thus explain the differences seen by eNose. In PCD, these metabolites may be comprised of inflammatory cytokines such as interleukin (IL)-8 in combination with lower DNA content in airway secretions compared to CF, as well as lower proteolytic enzyme levels [65,66]. The investigators also observed that pulmonary exacerbations altered exhaled breath profiles [64]. Both PCD and CF are diseases in which early diagnosis, frequent monitoring, and aggressive treatment of airway infections help preserve lung function over time [64,67]. Therefore, non-invasive techniques to detect or monitor respiratory infections are becoming increasingly important not only for patients unable to expectorate sputum due to younger age, but also for those on effective therapies such as cystic fibrosis transmembrane conductance regulator (CFTR) targeting drugs [68].

A few studies have explored the potential role of eNose in detecting airway colonization with pathogens. Fungal infections with *Aspergillus fumigatus* were identified by way of an eNose in studies by de Heer et al. [69]. In the setting of invasive pulmonary aspergillosis (IA) in patients with prolonged chemotherapy-induced neutropenia (PCIN), they initially showed that patients with PCIN and IA presented with characteristic exhaled breath profiles [69]. In a more recent study by the same group, using a Cyranose 320 they showed that *A. fumigatus* airway colonization in patients with CF also led to a distinct breathprint [70]. 27 CF patients, of whom nine were colonized with *A. fumigatus*, were correctly classified by eNose with a cross-validated accuracy of 89%. eNose data were analyzed using PCA, the factors of which were then used for linear canonical discriminant analysis (LCDA). Overall, eNose-generated breathprints of CF patients with and without *A. fumigatus* colonization were significantly different [70]. They highlighted the previously-identified in vitro biomarker specific for *A. fumigatus*-induced invasive disease and colonization, 2-pentylfuran by GC-MS analysis [70,71].

One of the most common opportunistic pathogens leading to chronic bacterial lung infections in CF is *Pseudomonas aeruginosa* (PA). Persistent PA infection is known to be associated with increased morbidity and mortality in patients with CF [72]. Distinct eNose breath profiles of chronic PA infection were reported in CF patients by Joensen et al. (sensitivity and specificity of 71.4% and 63.3%, respectively, and AUROC of 0.69) [73]. In this cross-sectional case-control study 64 patients with CF, 21 with PCD, and 21 healthy controls were included [73]. Breathprints of CF patients with and without chronic infections by other pathogens, including *Achromobacter xylosoxidans* or *Stenotrophomonas maltophilia*, were not different (AUROC of 0.59). Significant differences were also not found between breath profiles of PCD patients with or without chronic PA infection [73]. Findings by Robroeks et al. support these observations; here, CF patients with PA colonization were discriminated via GC-MS from non-colonized patients on the basis of 14 exhaled VOCs [35]. Based on these VOCs, 100% discrimination was achieved between the two groups [35]. This work validates the concept of PA-specific VOCs that can be screened for by VOC-sensing instruments such as eNoses or GC-MS. Of relevance to studies suggesting that eNoses might be able to detect PA or other infections in exhaled breath by pattern analysis, recent studies have shown that specific VOCs can also be identified in fluid samples obtained from airways of CF patients. Nasir et al. analyzed volatile molecules from CF bronchoalveolar lavage (BAL) fluid using two-dimensional GC-time-of-flight-MS [74]. Utilizing nine specific volatile molecules, PA-positive (*n* = 7) were distinguished from PA-negative (*n* = 53) BAL samples with an AUROC of 0.86. Similar results were seen for *Staph. aureus* (SA)-positive and -negative samples [74]. Finally, eNoses have also shown potential in detecting infection in people with non-CF bronchiectasis. This chronic respiratory disease that is increasingly recognized in Europe and the United States [75,76], is characterized by irreversible dilation of the bronchi and by chronic airway inflammation [77], and similar to CF, PA airway infection contributes to morbidity as well [78]. In a study of 73 clinically stable patients with bronchiectasis by Suarez-Cuartin et al., using a Cyranose 320, airway infection produced different breath profiles compared to uninfected, with an accuracy of 72.1% and AUROC of 0.75 [34]. Further, breath profiles from subjects infected with PA were different from other pathogens (accuracy of 89.2%, AUROC of 0.96), or no infection patients (72.7%, AUROC of 0.82). Thus, these findings suggest the potential of an eNose to identify specific bacterial airway infections such as PA, regardless of underlying disease.

To summarize, the currently published data suggest that eNoses may be able to distinguish between exhaled breath profiles of patients with CF, PCD, and bronchiectasis, and to detect certain infections with pathogens such as *A. fumigatus* and *P. aeruginosa* (PA) [34,64,69,70,73]. This has potential implications for transforming patient care in the near future by implementing eNoses at the clinical point-of-care for early and accurate detection of infections. Further studies using eNose in combination with technologies able to identify specific molecular markers, such as GC-MS, are needed to help improve current eNose technologies. This could be done by adding sensors for specific VOC compounds identified by GC-MS to the sensor arrays on an eNose, for more precise recognition of disease-specific VOC profiles, as discussed above [59]. Utilizing more sensor data-points (e.g., 158 sensors in the 4-eNose-platform vs. 32 in the Cyranose 320) is an alternative strategy [46].

## 3. Future Directions and Need for Future VOC-based Studies

Current results of detecting both respiratory and non-respiratory diseases by eNose, as well as specific infections in some conditions, are promising. Rapid improvements in eNose technologies may overcome their current limitations, as newer generations of eNoses are being upgraded with more advanced sensor technologies and data analysis systems [28]. With this, new areas of research may evolve. As an example, recent work has demonstrated the ability of the eNose to diagnose different types of interstitial lung diseases (including cryptogenic organizing pneumonia, idiopathic pulmonary fibrosis, and connective tissue disease-associated ILD) [79,80]. eNoses are also becoming increasingly utilized to detect biomarkers of various types of malignancies outside of the respiratory system, including colorectal cancer, and Barrett’s esophagus, the precursor to esophageal adenocarcinoma [81,82].

**Figure 1:**
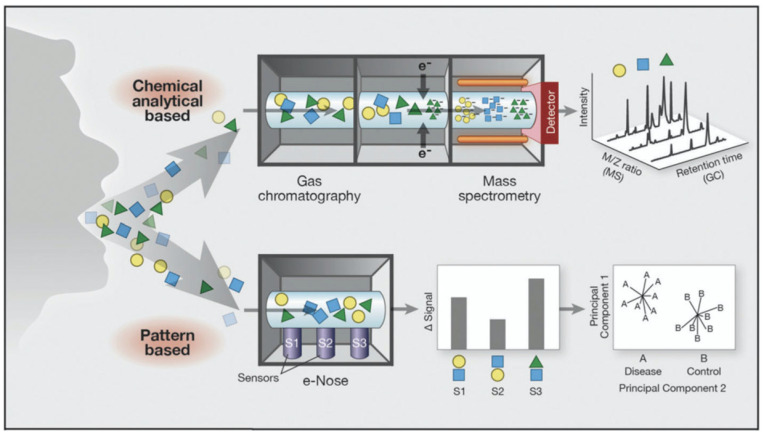
Complimentary analysis of volatile organic compounds by gas chromatography-mass spectrometry (GC-MS) (top) and eNose detection (bottom) [89]. GC-MS can be utilized to identify specific VOC biomarkers that make up eNose-detected breath profiles. Figures reproduced with permission [89].

Further, studies investigating VOC metabolomics have also yielded promising results in respiratory and non-respiratory conditions. By combining the eNose with GC-MS, detecting individual VOCs may not only improve sensitivity and specificity, but also allow for the detection of novel, previously unrecognized biomarkers and biological pathways (Figure 1). Several studies have already taken this approach. For example, Rodriguez-Aguilar et al. used an eNose coupled with GC-MS to identify and match specific VOCs to breathprints obtained by eNose from patients with COPD [83], identifying biomarkers of COPD in real time. VOC biomarkers of pulmonary oxygen toxicity have also become identifiable when combining eNose and GC-MS in a study of scuba divers by Wingelaar et al. [84]. Research in CF suggests that VOC breath profiles identify SA infection; by using GC-MS, breath VOC profiles were classified, and distinguished SA-infected and non-infected CF patients with 100% sensitivity and 80% specificity [85]. Potential biomarkers specific for SA detection are isovaleric acid and methylbutanal [86,87]. In the aforementioned study of *A. fumigatus* colonization of patients with CF by de Heer et al., GC-MS analysis was suggested to complement eNose testing [70]. Definitive exhaled biomarkers of *A. fumigatus* infection, including 2-pentylfuran as well as monoterpenes and sesquiterpenes have been identified [88]. It is possible that factors other than *A. fumigatus* metabolites such as host inflammatory responses to *A. fumigatus*, exposure to antimicrobial therapy or corticosteroids, or more-severe CF lung disease, also contribute to the VOC patterns detectable by eNose in these patients [70]. However, VOC breathprints detectable by eNose seem to be disease-specific as inflammatory airway diseases such as asthma, COPD, CF, and PCD can all be discriminated by this technology [41,50,64,73]. 

## 4. Conclusions

Taking advantage of detecting VOCs exhaled in human breath, eNose technology has enormous potential to improve or offer alternative solutions to current diagnostic tests for respiratory diseases. eNoses provide increasingly accurate and sensitive discriminative power to help differentiate between health and disease, sub-types of diseases and also disease activity and control (Table 1). In addition, eNose technology may represent a non-invasive tool to detect infections as they occur in patients with respiratory diseases including lung malignancies, asthma, COPD, CF, and PCD. While the eNose has the potential to be used as a screening tool at the clinical point-of-care, its integration with specific analytic methods such as GC-MS will help identify new biomarkers of disease and disease control.

## Figures and Tables

**Table 1:** Summary of key studies presented in this review.

Publication [Ref.]	Disease	Number of Patients/Total Study Participants	Type of VOC Detection Device	Main Findings
Tirzīte et al. [38]	Lung cancer	252/475	Cyranose 320	High-risk controls vs. cancer Sensitivity: 96%, specificity: 92% Non-smokers vs. cancer Sensitivity: 96%, specificity: 91%
Van de Goor et al. [9]	Lung cancer	52/144	Aeonose	High-risk controls vs. cancer Sensitivity: 83%, specificity: 84%
McWilliams et al. [26]	Lung cancer	25/191	Cyranose 320	High-risk controls vs. cancer Sensitivity: 81.3%, specificity: 88%
Rocco et al. [11]	Lung cancer	23/100	BIONOTE eNose	High-risk controls vs. cancer Sensitivity: 86%, specificity: 95%
Lamote et al. [40]	Lung cancer (malignant pleural mesothelioma, [MPM])	35/64 (19 asymptomatic asbestos-exposed subjects [AEx] + 16 control, 14 MPM, 15 benign disease [ARD])	Common Invent, Owlstone Lonestar, Cyranose 320, Tor Vergata eNoses + GC-MS	AEx subjects vs. MPM eNose: 97% accuracy GC-MS: 97% accuracy MPM vs. AEx + ARD eNose: 74% accuracy GC-MS: 94%
De Vries et al. [16]	Lung cancer	143/143	SpiroNose	Responders vs. Non-responders to anti-PD-1 therapy Sensitivity: 81%, specificity: 50%
Dragonieri et al. [41]	Asthma	20/40	Cyranose 320	Mild asthma vs. young controls Cross-validation: 100% Severe asthma vs. old controls Cross-validation: 90%
Plaza et al. [42]	Asthma	52/52	Cyranose 320	Eosinophilic vs. neutrophilic Accuracy: 73%, AUROC: 0.92 Eosinophilic vs. paucigranulocytic Accuracy: 74%, AUROC: 0.79 Neutrophilic vs. paucigranulocytic Accuracy: 89%, AUROC: 0.88
Brinkman et al. [46]	Asthma	78/78	Common Invent, Owlstone Lonestar, Cyranose 320, Tor Vergata eNoses	Inflammatory phenotypes in severe asthma Three distinct clusters (*n* = 26, *n* = 33, *n* = 19)
Van der Schee et al. [45]	Asthma	25/45	Cyranose 320	Asthma vs. controls Sensitivity: 80%, specificity: 65%
Tenero et al. [47]	Asthma	28/38	Cyranose 320	Non-symptomatic asthma (control + controlled asthma) vs. symptomatic asthma (partially controlled + uncontrolled asthma) Sensitivity: 0.79, specificity: 0.84
Montuschi et al. [36]	Asthma	27/51	Tor Vergata eNose + GC-MS	Asthma vs. controls eNose: 87.5% accuracy GC-MS: “significantly different”
Brinkman et al. [51]	Asthma	23/23	Common Invent, Owlstone Lonestar, Cyranose 320, Tor Vergata eNoses + GC-MS	eNose: baseline vs. loss of control: 95% accuracy loss of control vs. recovery: 86% GC-MS: baseline vs. loss of control: 68% accuracy loss of control vs. recovery: 77%
Bannier et al. [48]	Asthma & CF	33/55 (20 asthma, 13 CF)	Aeonose	Asthma vs. CF Sensitivity: 0.89, specificity: 0.77 CF vs. controls Sensitivity: 0.85, specificity: 0.77 Asthma vs. controls Sensitivity: 0.84, specificity: 0.91
Fens et al. [50]	Asthma & COPD	60 asthma, 40 COPD	Cyranose 320	COPD vs. fixed asthma Sensitivity: 85%, specificity: 90% COPD vs. classical asthma Sensitivity: 91%, specificity: 90%
Shafiek et al. [33]	COPD	143/173 (90 Exacerbated COPD [ECOPD], 50 stable COPD [SCOPD])	Cyranose 320	SCOPD vs. controls Sensitivity: 72%, specificity: 70% ECOPD vs. controls Sensitivity: 66%, specificity: 80% ECOPD vs. SCOPD Sensitivity: 89%, specificity: 48%
Finnamore et al. [58]	COPD	63/63	BIONOTE eNose	BODE functional status predicted via eNose Sensitivity: 0.71, specificity: 0.93
Van Velzen et al. [59]	COPD	31/68 (31 ECOPD, 37 COPD)	Common Invent, Owlstone Lonestar, Cyranose 320, Tor Vergata eNoses + GC-MS	ECOPD vs. COPD eNose: Accuracy: 75% GC-MS: Accuracy: 71%
Paff et al. [64]	CF & PCD	50/73 (25 CF, 25 PCD)	Cyranose 320	CF vs. controls Sensitivity: 84%, specificity: 65% PCD vs. controls Sensitivity: 88%, specificity: 52% CF vs. PCD Sensitivity: 84%, specificity: 60%
Joensen et al. [73]	CF & PCD	85/106 (64 CF, 21 PCD)	Cyranose 320	CF with *P. aeruginosa* (PA) vs. CF without PA Sensitivity: 71.4%, specificity: 63.3% No sig. difference between: CF with non-PA infection vs. CF without infection & PCD with PA/other infection vs. PCD without infection
De Heer et al. [70]	CF	27/27	Cyranose 320	CF with (*n* = 9) and without (*n* = 18) *A. Fumigatus* Sensitivity: 78%, specificity: 94%
Suarez-Cuartin et al. [34]	Bronchiecta-sis	73/73	Cyranose 320	Bronchiectasis with PA vs. Bronchiectasis without PA Sensitivity: 92%, specificity: 85%

*A. fumigatus:* Aspergillus fumigatus, AEx: asymptomatic former asbestos-exposed, ARD: benign asbestos-related diseases, AUROC: area under the receiver operating characteristic curve, BODE: body mass index, airflow obstruction, dyspnea, and exercise, CF: cystic fibrosis, COPD: chronic obstructive pulmonary disease, ECOPD: exacerbated COPD, eNose: electronic nose, GC-MS: gas chromatography-mass spectrometry, MPM: malignant pleural mesothelioma, PA: Pseudomonas aeruginosa, PCD: primary ciliary dyskinesia, PCIN: prolonged chemotherapy-induced neutropenia, SCOPD: stable COPD, U-BIOPRED: Unbiased biomarkers for the prediction of respiratory disease outcomes, VOCs: volatile organic compounds.

## Abbreviations

AEx	Asymptomatic former asbestos-exposed subjects
ARD	Benign asbestos-related diseases
AUROC	Area under the receiver operator characteristic
BAL	Broncho-alveolar lavage
BODE	Body mass index, obstruction, dyspnea, and exercise
CD	Crohn’s disease
CF	Cystic fibrosis
CFTR	Cystic fibrosis transmembrane conductance regulator
COPD	Chronic obstructive pulmonary disorder
CVV	Cross-validation value
ECOPD	Exacerbations of COPD
eNose	Electronic nose
GC-MS	Gas chromatography-mass spectrometry
IA	Invasive pulmonary aspergillosis
IBD	Inflammatory bowel disease
LCDA	Linear canonical discriminant analysis
LRA	Logistic regression analysis
MPM	Malignant pleural mesothelioma
PA	*Pseudomonas aeruginosa*
PCA	Principal component analysis
PCD	Primary ciliary dyskinesia
PCIN	Prolonged chemotherapy-induced neutropenia
PLS-DA	Partial least square discriminant analysis
POC	Point of care
PPMs	Potential pathogenic micro-organisms
SA	*Staphylococcus aureus*
TB	*Mycobacterium tuberculosis*
UC	Ulcerative colitis
VOCs	Volatile organic compounds
6MWD	Six-minute walk test distance
